# Indomethacin-Enhanced Anticancer Effect of Arsenic Trioxide in A549 Cell Line: Involvement of Apoptosis and Phospho-ERK and p38 MAPK Pathways

**DOI:** 10.1155/2013/237543

**Published:** 2013-11-10

**Authors:** Ali Mandegary, Maryam Torshabi, Mohammad Seyedabadi, Bagher Amirheidari, Elham Sharif, Mohammad Hossein Ghahremani

**Affiliations:** ^1^Neuroscience Research Center, Institute of Neuropharmacology, Kerman University of Medical Sciences, Kerman 7619813159, Iran; ^2^Department of Pharmacology and Toxicology, Faculty of Pharmacy, Tehran University of Medical Sciences, Tehran 1417614411, Iran; ^3^Department of Dental Biomaterial, Dental School, Shahid Beheshti University of Medical Sciences, Tehran 1473775543, Iran; ^4^Department of Molecular Imaging, The Persian Gulf Biomedical Sciences Research Institute, Bushehr University of Medical Sciences, Bushehr 7514633341, Iran; ^5^Pharmaceutics Research Center, Institute of Neuropharmacology, Kerman University of Medical Sciences, Kerman 76175493, Iran; ^6^Department of Molecular Medicine, School of Advance Technologies in Medicine, Tehran University of Medical Sciences, Tehran 1417755469, Iran; ^7^Basic and Clinical Toxicology Research Centre, Faculty of Pharmacy, Tehran University of Medical Sciences, Tehran 1417614411, Iran

## Abstract

*Background*. Focusing on novel drug combinations that target different pathways especially apoptosis and MAPK could be a rationale for combination therapy in successful treatment of lung cancer. Concurrent use of cyclooxygenase (COX) inhibitors with arsenic trioxide (ATO) might be a possible treatment option. *Methods*. Cytotoxicity of ATO, dexamethasone (Dex), celecoxib (Cel), and Indomethacin (Indo) individually or in combination was determined at 24, 48, and 72 hrs in A549 lung cancer cells. The COX-2 gene and protein expression, MAPK pathway proteins, and caspase-3 activity were studied for the most cytotoxic combinations. *Results*. The IC_50_s of ATO and Indo were 68.7 **μ**mol/L and 396.5 **μ**mol/L, respectively. Treatment of cells with combinations of clinically relevant concentrations of ATO and Indo resulted in greater growth inhibition and apoptosis induction than did either agent alone. Caspase-3 activity was considerably high in the presence of ATO and Indo but showed no difference in single or combination use. Phosphorylation of p38 and ERK1/2 was remarkable in the concurrent presence of both drugs. *Conclusions*. Combination therapy with ATO and Indo exerted a very potent in vitro cytotoxic effect against A549 lung cancer cells. Activation of ERK and p38 pathways might be the mechanism of higher cytotoxic effect of ATO-Indo combination.

## 1. Introduction

Health care practitioners face a huge burden caused by lung cancer. Lung cancer is also responsible for a considerable share of cancer-induced mortalities, which is estimated to exceed 800,000 annual deaths worldwide [1]. One clinical obstacle to successful cure of lung cancer is the resistance to chemotherapeutic agents. Besides, the side effects of drug regimens routinely employed in cancer treatment are associated with the treatment failure. One way to overcome these complications is to utilize multiple drugs enlisting various mechanisms to attack the disease [2]. Combination chemotherapy aimed at increasing cytotoxic efficacy has experienced a huge evolution over recent decades [2]. A shared mechanism of resistance towards a chemotherapeutic drug is the failure of the tumor cells to go into apoptosis. Hence, interest has been raised to exploit this path to circumvent drug resistance. 

This might justify the routine use of combination chemotherapy in the treatment programs of lung cancers. However, treatment efficacy has not always been promising even when recently formulated combinations were used. This has opened a window to explore the utilization of other agents which might not even be a part of current general cancer therapy strategies. Arsenic trioxide (ATO) is presently only used for the treatment of acute promyelocytic leukemia (APL) under the approval of FDA [3]. Application of ATO in combination with other agents in different cancers including lung could potentially provide an effective therapeutic tool. In addition, there are alternative mechanisms which can promote the apoptotic death of cancerous cells. A large body of evidence has shown a significant increase in expression of cyclooxygenase-2 (COX-2) in variety of cancers [4–6]. Thus, many COX inhibitors have been shown to prevent or delay development of certain tumors including lung cancer. It has been reported that nonsteroidal anti-inflammatory drugs (NSAIDs) including celecoxib and indomethacin can induce apoptosis in various types of lung cancer cells [7–10]. Furthermore, dexamethasone, a potent synthetic glucocorticoid which inhibits cyclooxygenase none selectively [11, 12], is being used in various cancer treatment protocols to prevent cancer progression and/or avoid side effects of chemotherapeutic agents [13–15]. However, lines of evidence of NSAIDs' antilung cancer effects due to the COX-2 inhibition are conflicting [9, 10, 16, 17]. 

It is notable that ATO can induce apoptosis in a variety of hematologic and solid tumor cancer cells, for which several mechanisms have been accounted [18–20]. Recently we have shown that ATO induces apoptosis and caspase-3 activation in the bone marrow promyelocytes of newly diagnosed APL patients through upregulation of p38 MAPK and Bax [21]. Despite the good therapeutic effects in APL even at low concentrations, the results obtained in non-APL patients have not been encouraging. For this reason enormous efforts have been made to provide effective combinations of ATO with other agents [22–24]. 

Therefore, this study aimed at investigating the effects of upstream, selective and nonselective COX inhibition, that is, dexamethasone, celecoxib, and indomethacin, respectively, on ATO-induced cell apoptosis in human lung cancer cell line. 

## 2. Materials and Methods

### 2.1. Reagents

Arsenic trioxide (As_2_O_3_; ATO) was obtained from Sina-Darou (Iran) in the stock concentration of 5 mM and then diluted with PBS to a working concentration of 50 *μ*M. Dithiothreitol (DTT), Tripure, Western blot detection kit, XTT assay kit, and polyvinylidene difluoride (PVDF) membrane were from Roche Applied Science (Germany). Anti-extracellular receptor kinase 1/2 (ERK1/2), phospho-ERK1/2, p38, phospho-p38, jun N-terminal kinase (JNK), Akt, Bax, COX-2, and *β*-actin antibodies were purchased from Cell Signaling Technology (USA). Bromophenol Blue, Coomassie blue R-250, and caspase-3 colorimetric assay kit were purchased from Sigma Chemical Company (UK). RPMI-1640, Fetal Bovine Serum (FBS), penicillin-streptomycin, and trypsin-EDTA were purchased from Bio-Sera (Korea). BioMax film was obtained from Kodak (UK). M-MuLV, primers, dNTPs, and Taq DNA polymerase were from Fermentas Life Science (Lithuania). Indomethacin (Indo) and Celecoxib (Cel) were kindly provided by a collaborative lab (as 98.8% purity) [25] and dissolved in minimal amounts of dimethyl sulfoxide (DMSO) so that the final DMSO in test wells did not exceed 1%. All other chemicals were from Merck (Germany).

### 2.2. Cell Culture

The A549 cell line (human lung adenocarcinoma epithelial cells) was cultured at 1 × 10^5^–1 × 10^6^ cells per mL in RPMI 1640 medium supplemented with 10% FBS, 100 U/mL penicillin, and 100 mg/mL streptomycin in a humidified atmosphere with 5% CO_2_. All the treatments were carried out on cells in logarithmic phase of growth. 

### 2.3. XTT Assay for Cellular Proliferation

Cells (7,000 cells per well) were seeded into 96-well flat-bottomed microplates and let adhere onto the surface. ATO (2–150 *μ*M), Indo (2–800 *μ*M), Cel (5–150 *μ*M), and Dex (10–200 *μ*M) and their combinations (at concentrations indicated in the graphs) were added to the wells. To measure viability, 50 *μ*L fresh XTT labeling mixture (49 *μ*L XTT labeling reagent and 1 *μ*L electron coupling reagent) was added after 24, 48, and 72 hrs and incubated for 4 hrs at 37°C in 5% CO_2_ atmosphere. Cell viability was assayed in quadruplicate, and the whole experiments were repeated three times. Absorbance at 450 nm (A_450 nm_) of the formazan was measured using a microplate reader, and the results were expressed as a ratio of the treated cells over the untreated vehicle cells. Solvent control trials were performed appropriately and exhibited no cytotoxic effects.

### 2.4. Protein Isolation and Western Blotting

Cell extracts were prepared by lysing the ice-cold PBS washed cells in the designated times by using 100 *μ*L lysis buffer (50 mM HEPES, pH 7.4, 5 mM CHAPS, 5 mM DTT) at 4°C for 15 min. Extracts were then centrifuged at 14,000 g in a microfuge at 4°C, and supernatants were transferred to fresh tubes. Protein concentration was determined by Bradford assay method. Thirty *μ*g of proteins was added to an equal volume of 2X SDS-sample buffer and run on a 10% sodium dodecyl sulfate-polyacrylamide gel (SDS-PAGE). Proteins were transferred to PVDF membranes (Roche), stained with 0.1% Ponceau S to ensure equal protein loading, and blocked with 1% casein in TBS (50 mM Tris, 150 mM NaCl) for 1 hour at room temperature. The membranes were then hybridized overnight at 4°C with polyclonal anti-Bax (1 : 2000), anti-Cox-2, anti-p38 (1 : 1000), anti-ERK1/2 (1 : 1000), anti-JNK (1/1000), anti-Akt (1/1000), anti-phospho-ERK, anti-phospho-p38, and anti-*β*-actin (1 : 1000) antibodies (Cell Signaling, USA). Following 2 washes with TBS-T (TBS containing 0.1% Tween-20) and one wash with TBS for 12 minutes each, blots were incubated with a goat anti-mouse/rabbit-antibody-HRP conjugate (Roche) for 1 hr at room temperature. After washing the blots as previously, bands were visualized by adding luminal substrate to the blots and exposure to the BioMax film (Kodak).

### 2.5. Isolation of Total Cellular RNA, cDNA Synthesis, and RT-PCR

RNA was isolated from log phase cultures of the cell lines. Cells were harvested by centrifugation at 110 g for 5 min and washed with PBS and total cellular RNA was extracted using TRIPURE reagent (Roche, Germany) following the manufacturer's instruction. RNA concentration and its purity were estimated by measuring the absorbance at 260 and 280 nm. Isolated RNAs were stored at –80°C and subsequently used for semiquantitative RT-PCR. Single stranded cDNA was synthesized using M-MuLV Reverse Transcriptase (Fermentas, Lithuania) and random hexamer primers according to the manufacturer's protocol. 

For RT-PCR 1 *μ*g of cDNA was used as the target in a total volume of 25 *μ*L. Reactions were performed according to standard protocols using the following primers and conditions: for Cox-2 (GenBank: AJ634912.1) forward 5′-AGC TGG GAA GCC TTC TCT AAC-3′ and reverse 5′-AGA TCA TCT CTG CCT GAG TAT CTT-3′ and for *β*-actin (as internal control; GenBank: X00351) forward 5′-GAT GAT GAT ATC GCC GCG CT-3′ and reverse 5′-CTT CTC GCG GTT GGC CTT GG-3′ primers were used. PCR condition for COX-2 and *β*-actin was 35 cycles of denaturation at 94°C for 30 sec, annealing at 55°C for 30 sec, and extension at 72°C for 30 sec. PCR products were electrophoresed on a 1.5% agarose gel containing 500 *μ*g/L ethidium bromide and visualized with UV light. The amplified bands for COX-2 and *β*-actin were 302 and 351 bp, respectively. All experiments were performed three times. 

### 2.6. Caspase-3 Activity Assay

Activity of caspase-3 was assayed according to the manufacture guideline (Sigma). Briefly, 30 *μ*g of lysate, 90 *μ*L of assay buffer (20 mM HEPES, pH 7.4, 0.1% CHAPS, 5 mM DTT, 2 mM EDTA) and 10 *μ*L of caspase-3 substrate Ac-DEVD-pNA (2 mM) were added to each well of a flat-bottom 96 well. As positive control, the caspase-3 pure protein was used. For measuring the nonspecific hydrolysis of the substrate, an inhibitor-treated cell lysate control was included. Microplate was incubated at CO_2_ incubator and O.D. 405 nm was measured in 4 hrs (in this time there was the biggest difference).

### 2.7. Statistical Analysis

Statistical analysis was done using SPSS software (version 11.0). Data were expressed as Mean ± SD. One-way analysis of variance (ANOVA), followed by the Tukey HSD, was used to assess significant differences between treatment groups. Differences were considered as significant when *P* < 0.05. The IC_50_ was calculated using Probit command in the SPSS.

## 3. Results

### 3.1. Arsenic Trioxide Inhibits the Growth of A549 Cells Dose- and Time-Dependently

**Figure 1:**
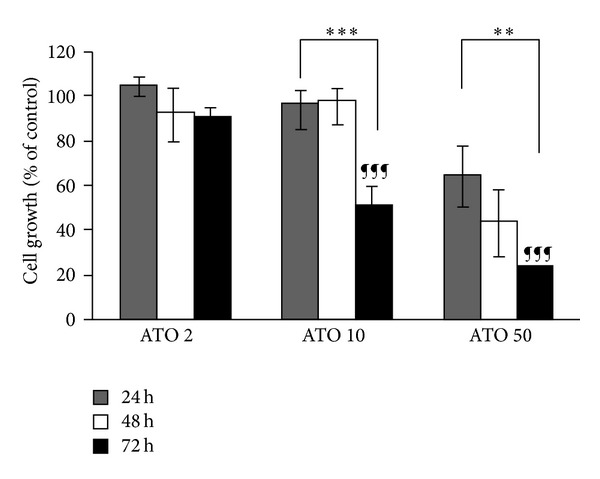
Effect of different incubation time on the cytotoxicity of ATO on the A549 cell line (Mean ± SE, *n* = 4; **P < 0.01 and ***P < 0.001; ^¶¶¶^ *P* < 0.001 compared to ATO 2 *μ*M).

**Figure 2:**
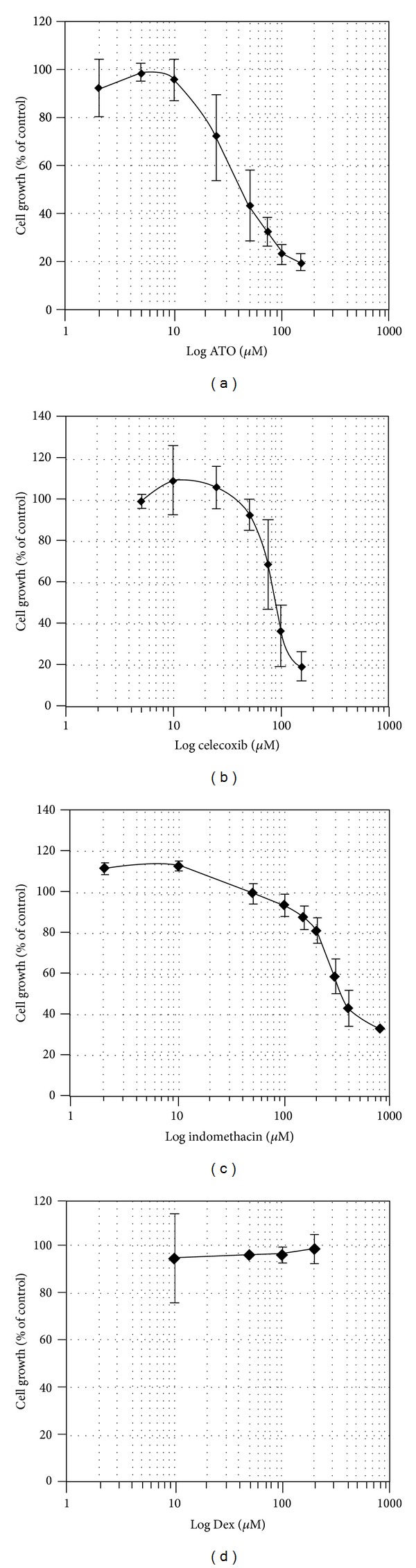
Effect of different concentrations of ATO (a), Cel (b), Indo (c), and Dex (d) on the growth of A549 cell line (Mean ± SE, *n* = 4).

ATO-induced inhibition of cell growth in A549 is illustrated in Figure 1. To explain further, a decrease in viability along with dose increments and time extensions is evident. Since the 24 and 48 hrs treatments had no significant cytotoxicity, assays were focused on the 72 hrs treatment. The dose-response curve of ATO, Cel, Indo, and Dex on cell proliferation at 72 hrs is shown in Figure 2. All the drugs, except Dex, induced cytotoxicity in a dose-dependent manner at 72 hrs. The calculated IC_50_s (concentration causing 50% growth inhibition) of agents were 68.7, 98.2, and 396.5 *μ*M for ATO, Cel, and Indo, respectively. Dex did not inhibit cell growth up to 200 *μ*M (Figure 2).

### 3.2. Arsenic Trioxide in Combination with Indo, and Not Dex or Cel, Produces More Potent Growth Inhibition Than with Either Agent Alone

**Figure 3:**
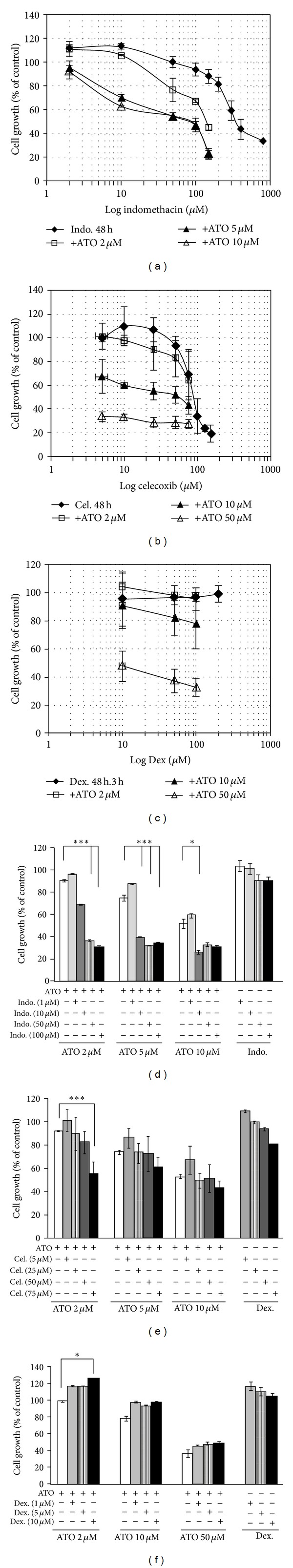
Inhibitory effects of single (a–c) and combination (d–f) of ATO and Indo, Cel and Dex on A549 lung cancer cell proliferation (Mean ± SE, *n* = 4; *P < 0.05, **P < 0.01 and ***P < 0.001).

Because ATO, Cel, and Indo had fairly high IC_50_ as single agent, we hypothesized that combination of these drugs would be more efficient to suppress the growth of the cells. We also tested the effect of Dex on the cytotoxic effect of ATO. Thus, cells were treated with combinations of Indo (1, 10, and 50 *μ*M), Cel (5, 25, 50, and 75 *μ*M), Dex (1, 5, and 50 *μ*M), and 2, 5, and 10 *μ*M concentrations of ATO for 72 hr and cytotoxicity was measured. As shown in Figure 3(a), Indo has no cytotoxic effect at 1, 10, and 50 *μ*M. However, combinations of noneffective doses of ATO (2, 5 *μ*M) and Indo (1, 10 *μ*M) have significantly decreased the viability of A549 cells. When cells are treated with the combination of ATO 2 *μ*M and Indo 10 *μ*M, the viability is reduced by 30% (Figure 3(d)). Indo 10 *μ*M has significantly augmented the antiproliferative effect of ATO 5 and 10 *μ*M to 60% and 80%, respectively (***P < 0.001 and *P < 0.05, compared to ATO alone). These results suggest that noneffective dose of Indo (10 *μ*M) has increased the cytotoxicity of low doses of ATO synergistically. Compared to ATO alone, combination of Indo 50 *μ*M with all the concentrations of ATO significantly decreased the cell proliferation (***P < 0.001) and Indo 50 *μ*M induced maximum effect on ATO toxicity. Interestingly a combination of Indo 50 *μ*M and ATO 5 or 10 *μ*M generates similar growth inhibition to Indo 800 *μ*M alone and 50–100 percent higher than ATO alone.  Figure 3(b) shows the effect of combination of ATO and Cel on A549 proliferation. Cel has reduced cell proliferation by 20% at 75 *μ*M (Figure 3(b)); however, this effect is not significant compared to control. When various doses of Cel are combined with ATO, the cytotoxicity is similar to ATO alone. Although the proliferation is reduced in the combination of ATO and Cel, the changes are not significantly different from ATO alone. ATO at low doses (2, 5 *μ*M) has increased celecoxib 75 *μ*M inhibitory effect and the ATO-Cel combination exhibits an additive effect (Figures 3(b)–3(e)). Combination of Dex and ATO treatment did not alter the antiproliferative effect of ATO in A549 cells (Figures 3(c)–3(f)). Even in low doses of ATO, cotreatment with Dex slightly increased the proliferation of cells.

### 3.3. ATO Decreases the Expression of COX-2 mRNA Dose-Dependently

**Figure 4:**
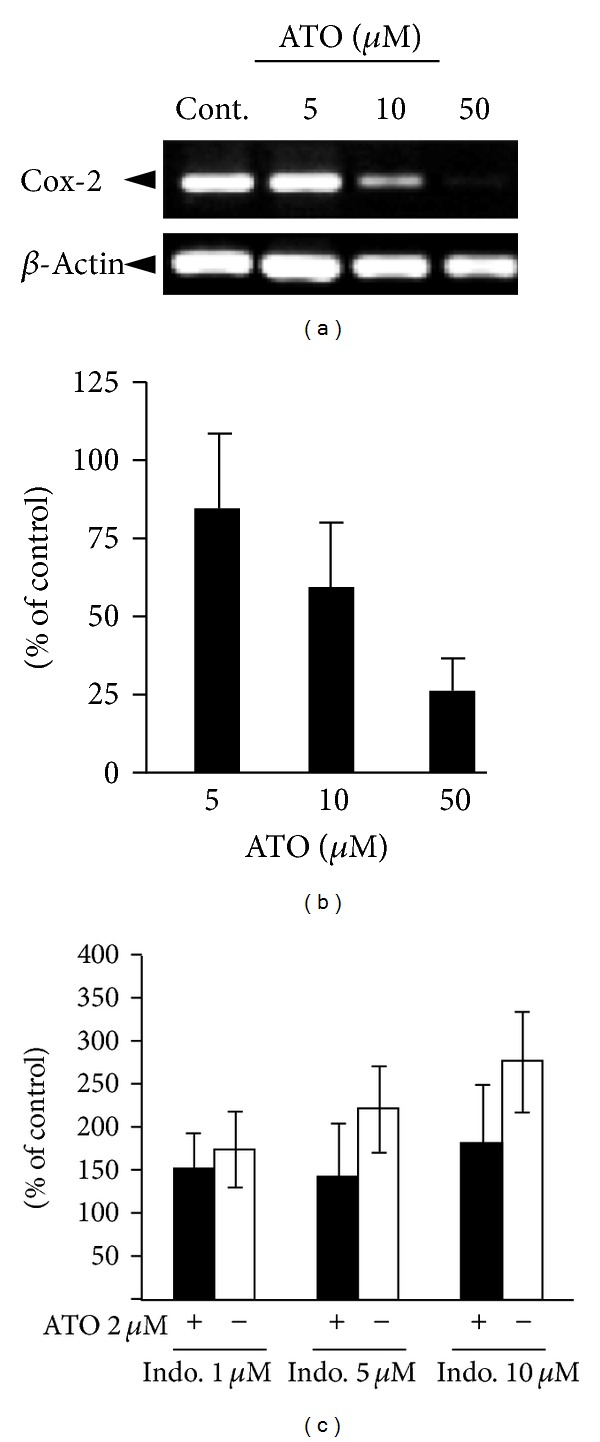
The effects of ATO and Indo and their combinations on the expression of COX-2 mRNA. (a) RT-PCR reaction products were resolved on 1% agarose gel and stained with Ethidium bromide. (b) Densitometric analyses of COX-2 mRNA expression is presented as the band's density to control (*β*-actin) of three independent experiments (Mean ± SE, *n* = 3). (c) The effect of Indo alone (light columns) and combination with ATO 2 *μ*M (dark columns) on COX-2 expression.

Considering the role of COX-2 and COX inhibition in lung cancer [26], we have assessed the mRNA expression of COX-2 with different concentrations of ATO as well as ATO 2 *μ*M combination with Indo.  Figure 4(a) shows that ATO has decreased the COX-2 expression in a dose-dependent manner (Figures 4(a) and 4(b)) and at ATO 10 *μ*M there is 50% reduction in COX-2 expression. Treatment of A549 cells with Indo has increased the COX-2 expression dose-dependently (Figure 4(c)). The Indo-induced COX-2 expression has been inhibited by addition of ATO 2 *μ*M to the cells (Figure 4(c)). 

### 3.4. Expression of Cox-2, Akt, ERK1/2, p38, JNK, and Bax Proteins in the Cells Treated with ATO, Indo, Dex, ATO/Indo, and ATO/Dex Combinations

**Figure 5:**
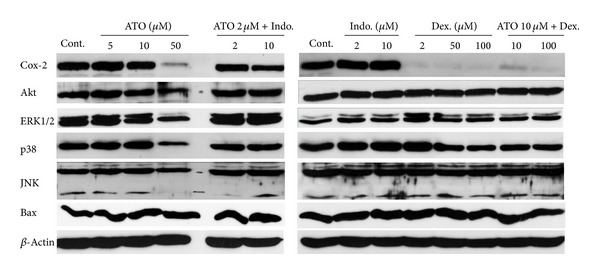
Western blot analysis of COX-2, Akt, ERK1/2, p38, JNK, and Bax proteins in A549 cells treated with ATO, Indo, Dex, ATO + Indo, and ATO + Dex combinations.

To address the role of proteins involved in the apoptosis and survival, the expression of Akt, ERK1/2, p38, JNK, and Bax proteins was determined by western blotting analysis. The expression of COX-2 protein decreased dose-dependently by ATO especially in the dose of 50 *μ*M (Figure 5). Indo alone did not change the expression of COX-2 protein. However, combination of ATO 2 *μ*M and Indo (2 and 10 *μ*M) decreased the COX-2 protein expression. ERK1/2 and p38 proteins levels were decreased with 50 *μ*M ATO treatment but remained unchanged with other treatments. Akt, Bax, and JNK seemed to be unchanged with different treatments.

Dex alone and in combination with ATO decreased expression of COX-2 protein completely. Furthermore, Dex decreased p38 and ERK1/2 proteins expressions dose-dependently which remained unaltered in combination with ATO.

### 3.5. ERK and p38 Proteins Were Highly Phosphorylated in the Cells Treated with ATO/Indo Combination

**Figure 6:**
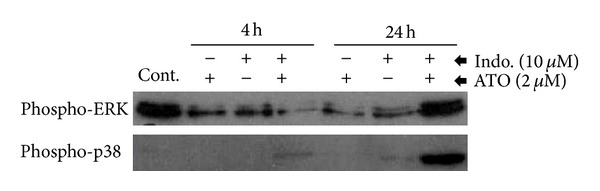
Phosphorylation of p38 and ERK in A549 cells treated with ATO, Indo, and ATO/Indo combination.

Since the change in the total ERK and p38 protein expressions was remarkable, we investigated the phosphorylation of ERK and p38 proteins in the ATO, Indo and ATO/Indo treatments. As shown in Figure 6, treatment of A549 cells with ATO and Indo alone lowered the phospho-ERK at 24 hrs; however, in cells treated with both ATO/Indo, the phosphorylation of ERK was increased and reached maximum level at 24 hr. Phosphorylation of p38 did not change in ATO and Indo single treatments. However, combination of ATO/Indo induced phosphorylation of p38 at 4 hrs and increased phospho-p38 to a remarkable level at 24 hr, suggesting a synergistic effect of combination treatment on p38 pathway activation.

### 3.6. Both ATO and Indo Activate Caspase-3

**Figure 7:**
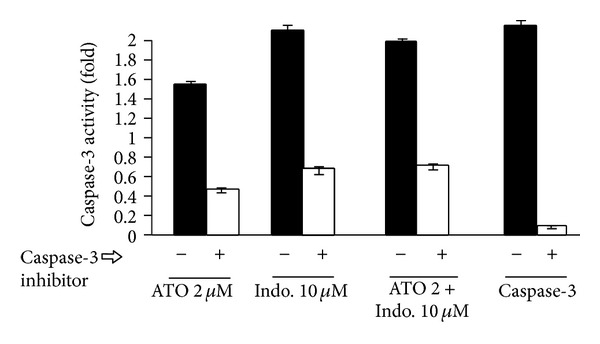
Activation of caspase-3 in A549 cells treated with ATO, Indo, and ATO/Indo combination.

To address the role of caspase-3 in the cytotoxicity of ATO, Indo, and ATO/Indo combination, the caspase-3 activity was measured. As shown in Figure 7, caspase-3 activity increased 1.2 and 1.6 fold with ATO 2 *μ*M and Indo 10 *μ*M, respectively. Increase in the caspase-3 activity in the cells treated with combination of ATO 2 *μ*M and Indo 10 *μ*M was similar to that of Indo 10 *μ*M. Caspase-3 inhibitor-treated cell lysate control showed that perhaps some other caspases are being activated in the treated cells. 

## 4. Discussion

Combination therapy with agents that hire different signaling pathways is a promising strategy for overcoming drug resistance in cancerous cells and for increasing treatment efficacy and/or decreasing drug toxicity [27]. The present findings indicate that Indo increases the cytotoxicity of ATO in human lung cancer cell line A549. This event is associated with activation of ERK and p38 MAPK signaling pathways. 

Our study showed that ATO, Cel, and Indo dose-dependently decreased cell viability (IC_50_s are 68.7, 98.2, and 396.5 *μ*M, resp.). Considering this effect, Indo produces very weak effect (IC_50_ = 396.5 *μ*M) compared to two other drugs. It has been shown that ATO mediates its cytotoxic effect by induction of apoptosis via disruption of mitochondrial transmembrane potentials and caspase-3 activation in various cell lines and human lymphocytes [18, 28]. Therefore, the cytotoxic effect of arsenic is related to apoptotic pathways since caspase-3 has been activated (Figure 7). On the other hand, accumulating body of evidence suggests that COX enzymes, especially COX-2, have been increased in many cancers including lung cancers [4, 6]. Thus, the inhibition of COX enzymes and in particular COX-2 can be accounted for the cytotoxic effect of Cel and Indo treatments. However, our results indicate a dramatic cytotoxicity with combinations of nontoxic doses of Cel or Indo with ATO in A549 cells (Figure 3). These results are of importance since very low doses of ATO and Indo (or Cel) have exerted significant cytotoxicity. The ATO/Indo combination treatment produced synergistic effect in all doses of ATO/Indo. Several combinations of NSAIDs with other chemopreventive drugs have previously been investigated in the lung cancers [22–24, 29, 30]. Lines of evidence show that anticancer effects of NSAIDs are not totally dependent on selectivity of COX-2 inhibition [9, 10, 31]. This perhaps justifies the difference in the observed effects of Indo/ATO and Cel/ATO combinations. In this regard, we have tested change in COX-2 expression (at mRNA and protein level) as well as several apoptosis-related proteins from MAPKs pathways as potential mechanisms for the observed enhanced cytotoxicity of indo/ATO combination. ATO dose-dependently has decreased COX-2 mRNA expression (Figure 4(a)); however at low dose (2 *μ*M), ATO has opposed the increase in COX-2 expression induced by Indo (Figure 4(c)). This is in consistent with the Han et al. [32] report that shows ATO decrease expression of COX-2. Besides, Dex has strong inhibitory effect on COX-2 expression (Figure 5) and little cytotoxic effect in these cells. When we evaluated the caspase-3 activation, ATO (2 *μ*M) and Indo (10 *μ*M) (as single or combination) have increased caspase-3 activation and therefore induced apoptosis. 

Changes in expression of the proteins of MAPK pathways have not been prominent except decreasing in p38, ERK1/2, and Akt proteins with ATO 50 *μ*M. However, activation of phospho-ERK and phospho-p38 is noticeable when combination of ATO 2 *μ*M and Indo 10 *μ*M has been used. These findings are in agreement with other studies that have reported activation of MAPK pathways with ATO and Indo alone [19, 21, 33]. MAPKs including ERK1/2, p38, and JNK have many important regulatory roles in the proliferation and apoptosis of the cells [34]. In general, JNK and p38 pathways are activated by stress stimuli and are involved in apoptosis, while ERK1/2 pathway is preferentially activated in response to growth factors [35]. Whereas ERK activation has generally been associated with antiapoptotic effects, activation of ERK has also been shown to be necessary for the cytotoxic-induced apoptosis [36]. We have previously shown that ATO treatment increases p38 and ERK expression in promyelocyte of APL patients [21]. Thus changes in MAPK pathways could be accounted for the ATO effect.

To our knowledge based on a search of literature no study has been conducted to assess the combination of Indo and ATO on the cancerous cells. Instead, in several studies the antiproliferative and apoptotic effects of sulindac, a structural isoform of indomethacin, have been studied. In a study on the NCI-H157 human lung cancer cells, Park et al. [37] showed that combination of ATO (2.5 *μ*M) and sulindac (5 *μ*M) increased apoptosis, reactive oxygen species (ROS), and oxidative stress, as evidenced by the heme oxygenase-1 (HO-1) expression and phosphorylation of ERK. Jin et al. [22] studied combination effect of ATO (1 *μ*M) and sulindac (200 *μ*M) on the H1299 human nonsmall cell lung carcinoma cells. They noted collapse of mitochondrial membrane potential and activation of caspase-3 in the cells cotreated with sulindac/ATO. Furthermore, the authors reported activation of JNK downstream of ROS generation. We here report phosphorylation of ERK in ATO/Indo treated cells and a strong activation of p38 kinase which can be involved in mediated stress induced cell death. However, ATO/Indo activated phosphorylation of p38 is in contrast with Jin et al. findings [22]. This may be due to cell type and/or involvement of this pathway in cytotoxicity.

The effect of low dose ATO/indo combination on cytotoxicity of A549 can be a multiple pathway function. It has been shown that indomethacin induces apoptosis in vivo [38] and in vitro in some cancerous cells including lung [7, 8], colon [39, 40], and leukemia [41]. In this report, production of ROS has been mentioned as the main mechanism of apoptotic effect [42, 43]. Besides, ROS also plays an important role in the ATO-induced apoptosis and cell death [28]. Consistent with this hypothesis, accumulating evidence indicates that L-buthionine-sulfoximine (BSO), a drug that depletes intracellular glutathione (GSH) and generates ROS, can sensitize the lung cancerous cells to ATO-induced cell death [24, 42, 44–46]. Moreover, ROS can activate ERK1/2 pathway [47] as well as p38 kinase [47, 48]. Thus, increase in p38 kinase and ERK activation besides change in COX-2 expression can be accounted for the ATO/indo induction of apoptosis in low doses.

## 5. Conclusion

In conclusion we here report a combination of low doses of ATO and indomethacin as strong inducer of apoptosis in A549. This finding is important since the doses of ATO and indomethacin are practically noneffective when used as single treatment. Thus, low dose combination may provide better treatment with less adverse effect in patient which needs to be studied in future.
